# Analysis of Human Factors Relationship in Hazardous Chemical Storage Accidents

**DOI:** 10.3390/ijerph17176217

**Published:** 2020-08-27

**Authors:** Wei Jiang, Wei Han, Jiankai Zhou, Zhishun Huang

**Affiliations:** 1 School of Emergency Management and Safety Engineering, China University of Mining & Technology (Beijing), Beijing 100083, China; zhoujk0607@163.com (J.Z.); huangzhishun730@126.com (Z.H.); 2 Chongqing Banan Port and Shipping Management Center, Chongqing 400054, China; hanwei2324@126.com

**Keywords:** hazardous chemical storage accidents, HFACS, chi-square test, odds ratio analysis, human factor relevance

## Abstract

Human factors are important causes of hazardous chemical storage accidents, and clarifying the relationship between human factors can help to identify the logical chain between unsafe behaviors and influential factors in accidents. Therefore, the human factor relationship of hazardous chemical storage accidents was studied in this paper. First, the human factors analysis and classification system (HFACS), which originated from accident analysis in the aviation field, was introduced. Since some items were designed for aviation accident analysis, such as the item “Crew Resource Management”, it is not fully applicable to the analysis of hazardous chemical storage accidents. Therefore, this article introduced some modifications and changes to make the HFACS model suitable for the analysis of hazardous chemical storage accidents. Based on the improved HFACS model, 42 hazardous chemicals storage accidents were analyzed, and the causes were classified. After analysis, we found that under the HFACS framework, the most frequent cause of accidents is resource management, followed by violations and inadequate supervision, and finally the organizational process and technological environment. Finally, according to the statistical results for the various causes of accidents obtained from the improved HFACS analysis, the chi-square test and odds ratio analysis were used to further explore the relevance of human factors in hazardous chemical storage accidents. The 16 groups of significant causal relationships among the four levels of factors include resource management and inadequate supervision, planned inappropriate operations and technological environment, inadequate supervision and physical/mental limitations, and technological environment and skill-based errors, among others.

## 1. Introduction

Hazardous chemical warehouses and storage tanks are locations for the storage and maintenance of hazardous chemicals such as chemical raw materials, chemical drugs, chemical reagents, pesticides, etc. Because of the large quantity, variety and high risk of hazardous chemicals, the potential storage hazards can exceed those of the production, transportation and use of hazardous chemicals. Human factors play an important role in the occurrence of hazardous chemical accidents in China [1,2]. Therefore, it is necessary to analyze and identify human factors in hazardous chemical storage accidents. In addition, this paper continued the research in reference [3], combining fault tree analysis (FTA) with human factors analysis and classification system (HFACS) model to analyze multiple hazardous chemical storage accidents. Based on this approach, we further explored the relationship between human factors in hazardous chemical storage accidents using the chi-square test and odds ratio analysis. According to the above methods, this paper realized the quantitative analysis of human factors.

It should be noted that the human factors studied in this paper not only refer to individual behaviors that directly lead to an accident, but also include other organizational factors such as organizational supervision and resource management, because individual people are not isolated and act as members of an organization. As a result, individual behavior is affected by other people, technology and the organization, and these factors restrict and influence each other. Therefore, the study of human factors should consider individual factors as well as organizational factors related to human behaviors.

Currently, certain human factors and proposed relevant models are available, including the software–hardware–environment–liveware (SHEL) model [4], Swiss cheese model [5] and the HFACS model [6]. Among these, the HFACS model has been widely recognized and adopted in many industries. Dekker noted that the HFACS model is the most powerful tool for human factors analysis of various accidents [7]. In the field of aviation, Shappell analyzed the data from 1020 aviation accidents in the United States and found that the majority of accidents were caused by the aircrew and environment, and the number of accidents related to supervision and organizational reasons was significantly reduced [8]. Daramola used the HFACS model to analyze aviation accidents in Nigeria and concluded that the most common causes of accidents were skill-based errors, the physical environment and inadequate supervision. Supervision violation to crew resource management to decision errors was considered the most likely path to accidents [9]. Michal et al. used the accident analysis method combining HFACS with a systems-theory accident model and processes (STAMP) to analyze the Überlingen air accident and confirmed the feasibility of the STAMP-HFACS analysis method [10]. Rashid et al. proposed the Human Factors Analysis and Classification System-Maintenance Extension (HFACS-ME) model for helicopter maintenance accidents and statistically analyzed 58 helicopter maintenance accidents to study the survival rate of helicopter maintenance accidents and the distribution of accident severity [11].

The application of HFACS in accident research also includes coal mining, maritime, medical, railway, chemical and other industries. For example, Patterson and Shappell used the HFACS-Mining Industry (HFACS-MI) model to analyze 508 coal mine accidents in Queensland and concluded that skill-based errors are the most common unsafe behavior, with no significant difference between different types of mines [12]. Chauvin et al. analyzed the human factors and organizational factors of ship collision accidents in Britain and Canada using the improved HFACS model [13]. The analysis showed that most collision accidents were caused by decision errors. Baysari et al. analyzed railway accidents in Australia using the HFACS and Technique for the Retrospective and Predictive Analysis of Cognitive Errors (TRACEr) methods and suggested the effectiveness of the two methods [14]. However, each tool seems to ignore certain important factors related to the occurrence of errors. Cohen et al. used HFACS-Healthcare to identify systemic vulnerabilities during surgery [15]. Hale et al. used the HFACS model to analyze 26 fatal building accidents and found deficiencies in planning and risk assessment, hardware design, purchase and installation, and contracting strategies [16]. In the chemical industry, Gong and Fan analyzed the “11·13” explosion accident at the PETROCHINA Jilin petrochemical biphenyl factory using HFACS and classified the human factors that led to the accident, confirming the usefulness and feasibility of the HFACS for accident analysis in the chemical industry [17]. Zhou et al. improved the items of HFACS and used the improved HFACS to analyze the “8·12” Tianjin Binhai New Area explosion accident [18]. That research showed that the interaction between different levels of human factors in the Ruihai company led to the accident, and the accident investigation report displayed limitations in the identification of human factors and guidance for similar accident prevention. A review of the main relevant information of the HFACS is shown in Table 1.

The HFACS model addresses the defects of the Reason model and gives a specific definition of the loopholes in each layer of the Reason model, which is more conducive to the study of the classification and mechanism of human factors. However, there are many reasons that exist for hazardous chemical storage accidents [2,19,20], and qualitative analysis of the causes of hazardous chemical storage accidents alone is not sufficient to ensure the effectiveness of the analysis results. Therefore, it is necessary to use other methods to quantitatively analyze the cause of an accident. For this reason, this paper collected information from 42 hazardous chemical storage accidents. The collected accident data were summarized, including the date of accident, enterprises, type of accident and number of deaths. The main sources of accident data include the national and local emergency management departments at all levels, the websites of local governments, the official website of the China Chemical Safety Association, and the chemical registration center of the Ministry of Emergency Management of the People’s Republic of China. Then, according to the characteristics of hazardous chemical storage accidents, the HFACS model was modified to make it more suitable for the analysis of hazardous chemical storage accidents. Second, the improved HFACS model was applied to accident analysis. In this way, the causes and high-frequency human factors of accidents under the HFACS framework were obtained. Finally, a chi-square test and odds ratio analysis were used to test the significance and relevance between the four levels of factors under the improved HFACS framework.

## 2. Accident Analysis Method

### 2.1. Improving the HFACS Model

Based on the Reason model, HFACS defines the dominant and implicit factors that cause accidents in the Reason model and describes four levels of human error: (1) Unsafe Acts, (2) Preconditions for Unsafe Acts, (3) Unsafe Supervision, and (4) Organizational Influences [6]. However, some items in the original HFACS framework might not correspond to the causes of hazardous chemical storage accidents, e.g., “Crew Resource Management”, “Routine Violation”, “Exceptional Violation” and other subcategories. Therefore, combined with the characteristics of hazardous chemical storage accidents, this paper made appropriate improvements to the original HFACS model. In this manner, a modified HFACS model was established which is more suitable for the analysis of hazardous chemical storage accidents.

**Figure 1:**
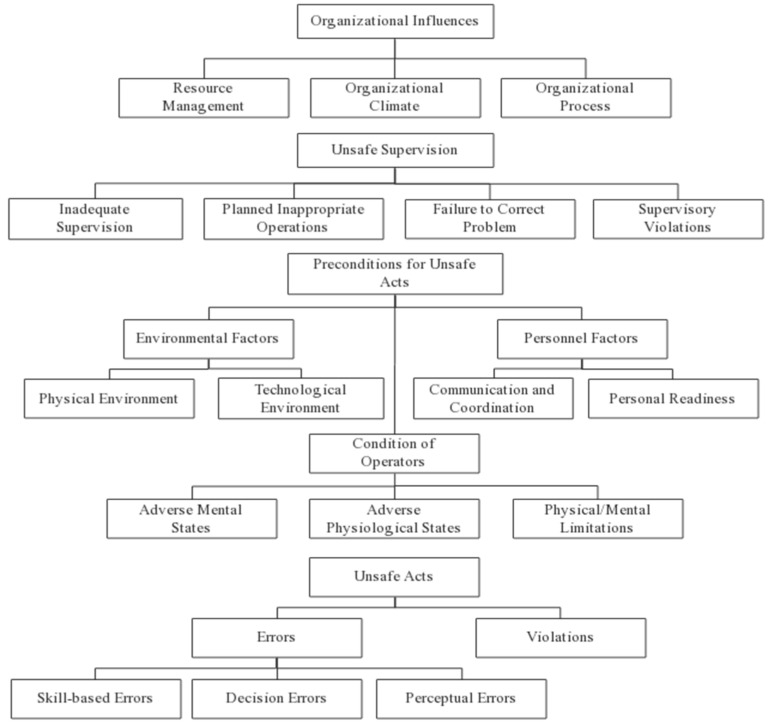
HFACS frame diagram of hazardous chemical storage accidents.

“Crew Resource Management” in the original HFACS model is changed to “Communication and Coordination”. Crew resource management is a professional term used in the field of aviation and usually refers to problems such as poor information communication and lack of team cooperation between the aircraft and air traffic control during task execution. Thus, in the HFACS, “Crew Resource Management” essentially refers to the problem of communication and coordination. In the storage of hazardous chemicals, if the information exchange among the superiors, subordinates, or employees of the enterprise is poor and the cooperation between teams is ineffective, unsafe behaviors also occur. Therefore, “Crew Resource Management” in the original HFACS model was changed to “Communication and Coordination”. In addition, from the hazardous chemical storage accident investigation report, it is impossible to determine whether front-line employee violations are “routine” or “exceptional”. As a result, this paper combined the two types of violations into one type: Violations. The specific meanings of the items in the HFACS model of hazardous chemical storage accidents are identified in Figure 1.

### 2.2. Chi-Square Test and Odds Ratio Analysis

After using the improved HFACS model to analyze the frequency of each accident cause, we used the chi-square test (*χ*^2^) and odds ratio analysis to analyze the relevance among the four levels of factors in the HFACS framework. In statistics, the *χ*^2^ test is often applied for relevancy testing of nonparametric data variables and the analysis of fixed type data. The odds ratio (OR) is used to measure the characteristic value of the relevance between occurrences of attribute A and attribute B in a specific group [21].

#### 2.2.1. Chi-Square Test

First, we used the *χ*^2^ test to analyze whether a significant causal relationship exists between different factors at the upper and lower levels of the improved HFACS model. The original hypothesis (H0) was proposed: there is no significant causal relationship between the upper and lower level factors in the improved HFACS model. The alternative hypothesis (H1) was also proposed: there is a significant causal relationship between the upper and lower level factors in the improved HFACS model. Because only two factors at a time are selected for correlation analysis, the relevant frequency statistics are calculated in the form of a 2 × 2 contingency table, and the *χ*^2^ value was calculated. The 2 × 2 contingency table for the calculation of the *χ*^2^ value is shown in Table 2.

In Table 2, *nij* represents the actual observed value, i.e., the actual statistical value. *fij* represents the theoretical observation value, which means the expected value under the assumption that the two variables are uncorrelated. It should be noted that the meanings of *n*_11_, *n*_12_, *n*_21_ and *n*_22_ are as follows: (1) if the tested high-level factors and low-level factors occur at the same time in an accident, it is recorded as one time, and the cumulative value is *n*_11_; (2) if the tested high-level factors in an accident do not appear but the low-level factors appear, it is recorded as one time, and the cumulative value is *n*_12_; (3) if the tested high-level factors in an accident appear but the low-level factors do not appear, it is recorded as one time, and the cumulative value is *n*_21_; (4) if the high-level factors and low-level factors are not found in an accident, it is recorded as one time, and the cumulative value is *n*_22_. In particular, for a 2 × 2 contingency table, if *A*, *B*, *C* and *D* represent the actual observation times *n*_11_, *n*_12_, *n*_21_ and *n*_22_ respectively in four cells, then the chi-square value can be calculated by the following formula:

(1) $$\chi^{2}=\frac{n{\left(AD-BC\right)}^{2}}{\left(A+B\right)\left(A+C\right)\left(B+D\right)\left(C+D\right)}$$

The *p* value can be obtained by looking up the value in the table when the degree of freedom (df) = 1. The *p* value has the following statistical significance: when *p* > 0.05, we should accept the original hypothesis (H0) and reject the alternative hypothesis (H1), and when *p* < 0.05, we should reject the original hypothesis (H0) and accept the alternative hypothesis (H1).

#### 2.2.2. Odds Ratio Analysis

For a 2 × 2 contingency table, the formula for calculating the *OR* value is given as follows:

(2) $$OR=\frac{AD}{BC}$$

and the relationship between the occurrence of upper factors and the occurrence of lower factors in the HFACS model is determined after obtaining the *OR* value: when the *OR* value is greater than 1, it indicates that the occurrence of upper factors in the HFACS model can increase the occurrence possibility of lower factors, and when the *OR* value is less than 1, it indicates that the occurrence of upper factors cannot increase the occurrence possibility of lower factors.

## 3. Analysis of Hazardous Chemical Storage Accidents

To more clearly show the causes of hazardous chemical storage accidents and their logical relationships with each other, we restored the development and evolution process of accidents using a combination of the fault tree analysis (FTA) method and the HFACS model to analyze the accidents in depth. The detailed analysis steps are given in the literature [3]. We used this method to analyze 42 hazardous chemical storage accidents. The details of the accidents are shown in Table 3.

After the analysis, the accident causes were classified and statistically analyzed. The frequency and percentage of each accident cause were obtained under the improved HFACS framework, as shown in Table 4.

## 4. Using χ^2^/OR to Analyze the Relevance of Human Factors in Hazardous Chemical Storage Accidents

To conduct a quantitative study of human factors in hazardous chemical storage accidents, the relevance among the factors in 42 accident cases was further studied based on the HFACS analysis. In this paper, a chi-square test and odds ratio analysis method were used to test the significance and relevance of four level factors in the improved HFACS framework, so as to realize the key step of human factor quantitative analysis.

Taking the calculation of the relevance between Communication and Coordination and Decision Errors in the improved HFACS model as an example, we calculated the *χ*^2^ value and *OR* value. Original hypothesis (H0): there is no significant causal relationship between Communication and Coordination and Decision Errors. Alternative hypothesis (H1): there is a significant causal relationship between Communication and Coordination and Decision Errors. Table 5 shows the statistical results of the frequency of occurrence in accidents. The *χ*^2^ value and *OR* value were calculated using Equations (1) and (2).

By calculation, *χ*^2^ = 4.582 and *OR* = 4.000 > 1, and by df = 1, we can obtain *p* = 0.032 < 0.05. Therefore, we rejected H0 and accepted H1. This means that there was a significant causal relationship between Communication and Coordination and Decision Errors. In addition, the *OR* value greater than 1 indicated that the occurrence of Communication and Coordination can increase the possibility of Decision Errors.

**Figure 2:**
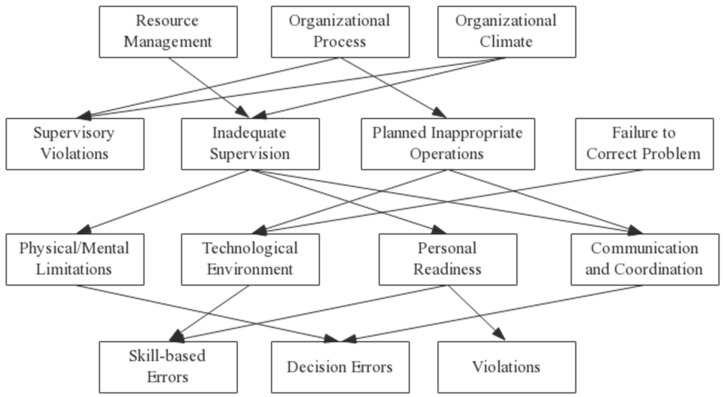
Cause and effect diagram of human factors in hazardous chemical storage accidents.

We used the above method to analyze the relevance among the four levels of factors in the improved HFACS model. We screened the causal relationships between different levels of factors that satisfy *p* < 0.05 and *OR* > 1, and eliminated the causal relationships of human factors that did not meet the conditions, e.g., Resource Management and Planned Inappropriate Operations, Organizational Climate and Failure to Correct Problem, Inadequate Supervision and Technological Environment, Failure to Correct Problem and Physical Environment, Personal Readiness and Perceptual Errors, etc. Thus, we obtained the results shown in Table 6. According to the inspection results, the causal relationship diagram of human factors in hazardous chemical storage accidents was obtained, as shown in Figure 2.

## 5. Results Analysis

According to the four levels of the improved HFACS framework (including Organizational Influences, Unsafe Supervision, Preconditions for Unsafe Acts, Unsafe Acts) and the results of the *χ*^2^ test and OR analysis, the results of quantitative calculation were analyzed as follows.

### 5.1. Defect of Organizational Influences

Organizational influences include Resource Management, Organizational Climate and Organizational Process. From Table 5, inadequate Resource Management has the greatest impact on Inadequate Supervision. In other words, if resource management of a hazardous chemical storage enterprise is poor, the possibility of insufficient supervision will increase to 20.7 times (*OR* = 20.667). Resource management loopholes are primarily reflected in human resources, equipment and facilities resources, funds, and other aspects. The specific performance issues are unreasonable personnel allocation, lack of a qualification examination system for special operation personnel, poor quality of safety management personnel, insufficient equipment and facilities, or quality defects. The poor organizational climate also leads to the occurrence of supervisory violations and inadequate supervision. A poor organizational climate will increase the possibility of Inadequate Supervision to 4.5 times (*OR* = 4.455) and the possibility of Supervisory Violations to 7.8 times (*OR* = 7.800). Poor organizational climate includes insufficient safety investment, insufficient risk management policies, “focusing on efficiency, ignoring safety”, and poor safety culture.

In addition, Organizational Process loopholes have a significant impact on Planned Inappropriate Operations and Supervisory Violations in Unsafe Supervision. Organizational process loopholes will increase the probability of Planned Inappropriate Operations to 6.2 times (*OR* = 6.231) and the probability of Supervisory Violations to 4.8 times (*OR* = 4.848). Organizational process loopholes are mainly reflected in systems, procedures, production supervision and other aspects. Examples of this include where the enterprise has not formulated a specific safety management system or the system is incomplete, the regulatory system has loopholes, the organization and management of the site are disordered, and relevant operation instructions are lacking.

### 5.2. Unsafe Supervision

Unsafe Supervision includes Inadequate Supervision, Planned Inappropriate Operations, Failure to Correct Problem and Supervisory Violations. It can be observed from Table 5 that Inadequate Supervision, Planned Inappropriate Operations, and Failure to Correct Problem in the Unsafe Supervision level have a significant impact on Technological Environment, Physical/Mental Limitations, Communication and Coordination, and Personal Readiness in the Preconditions for Unsafe Acts level. In particular, Inadequate Supervision can directly cause the emergence of three unsafe factors in the next level. If a hazardous chemical enterprise suffers from inadequate supervision, it will increase the possibility of worker physical or mental limitations to 5.1 times (*OR* = 5.143), the possibility of poor communication and coordination to 8.8 times (*OR* = 8.800), and the possibility of insufficient personal readiness to 14.3 times (*OR* = 14.286). Inadequate supervision refers primarily to situations in which the manager fails to offer sufficient training and guidance on hazardous chemical knowledge to the employees in their daily work. It results in employees lack of clarity relative to the physical and chemical properties and dangers of related hazardous chemicals or lack of supervision in fire operation sites. This situation further leads to poor information exchange and communication between the upper and lower levels of employees.

Planned Inappropriate Operations has a significant impact on the Technological Environment and Communication and Coordination in the Preconditions for Unsafe Acts level. Planned inappropriate operations will increase the possibility of a poor technological environment to 4.7 times (*OR* = 4.722) and poor communication and coordination to 4.3 times (*OR* = 4.333). Planned inappropriate operations is manifested as improper collocation between team members or authorization of unqualified team members for work, resulting in insufficient cooperation and communication among team members. In addition, planned inappropriate operations also refers to the improper allocation of resources, and thus it might lead to differences in equipment allocation among different teams, resulting in the risk of a poor technological environment. Failure to correct the problem will increase the probability of technological environmental problems to 4.3 times (*OR* = 4.275). Failure to correct the problem refers to the failure of the supervisor to find problems or correct the problems in time in the hazardous chemical storage process, resulting in the continuous existence of hazards. Poor technological environment refers to equipment and facility failures, lack of protective devices, lack of electronic monitoring facilities, unreasonable control design, etc. Therefore, it is easy to increase the risk of a poor technological environment if problems are not found or not solved in time, or hazards are not investigated adequately.

### 5.3. Preconditions for Unsafe Acts

Preconditions for Unsafe Acts includes the seven aspects of Physical Environment, Technological Environment, Adverse Mental States, Adverse Physiological States, Physical/Mental Limitations, Communication and Coordination, Personal Readiness, among others. However, for the 42 hazardous chemical storage accidents collected, only Technological Environment, Physical/Mental Limitations, Communication and Coordination, and Personal Readiness have a significant impact on Unsafe Acts. Among these, the lack of personal readiness is one of the main reasons for unsafe acts, especially for skill-based errors and violations. The lack of employee personal readiness can increase the probability of skill-based errors to four times (*OR* = 4.000) and increase the probability of violations to 13 times (*OR* = 13.000). Personal readiness refers to a lack of knowledge and skills for the related hazardous chemicals or a lack of physical strength and energy of the front-line workers before work. The main manifestations are insufficient knowledge about hazardous chemicals, a lack of mastery of skills required by the position, failure to wear personal protective equipment (PPE), insufficient rest, etc. Therefore, the lack of personal readiness will inevitably increase the possibility of skill-based errors and violations.

Physical/Mental Limitations and Communication and Coordination have a significant impact on the occurrence of Decision Errors. Physical or mental limitations can increase the probability of decision errors to 4.1 times (*OR* = 4.083). Communication and coordination can increase the probability of decision errors to 4 times (*OR* = 4.000). Physical/mental limitation refers to a lack of experience and the ability of employees to function in complex situations. Communication and coordination refers to insufficient cooperation among team members and lack of information exchange between superiors and subordinates. If these two factors are defective, it will inevitably lead to decision errors under different situations. Decision errors mainly refer to emergency judgment errors, emergency response errors, improper selection, problem handling errors, etc. In addition, the technological environment also has a significant impact on skill-based errors (*OR* = 9.000). A poor technological environment may lead workers to be unfamiliar with important equipment and ignore operational details, resulting in skill-based errors.

### 5.4. Unsafe Acts

Unsafe Acts includes Skill-based Errors, Decision Errors, Perceptual Errors and Violations. According to the statistics of unsafe acts in 42 hazardous chemicals storage accidents, violations by front-line workers account for the largest proportion, reaching 85.714%, followed by decision errors and skill-based errors, accounting for 52.381% and 47.619% respectively, and finally perceptual errors, accounting for 7.143%. Violations mainly refer to the violation of the existing rules and various safety operating procedures and risky operations of front-line workers.

Decision errors refer to the errors caused by improper employee estimation of the situation, including three main types of errors in emergency situations: process errors, selection errors and problem-solving errors. Skill-based error refers to mistakes in skill-related behaviors of employees, mainly including poor operation technology, blind operation blind, improper use of PPE, etc. Perceptual errors are manifested by individual cognition and actual conditions such as visual errors, information understanding errors, wrong directions, etc.

According to the above analysis, the main factors leading to unsafe acts of employees are personal readiness, communication and coordination, and technological environment, whereas unsafe acts are primarily reflected in violations and decision errors. Therefore, managers should enhance training on professional knowledge and skills for front-line operators, improve the professional development of employees, and assure good job preparation to reduce the possibility of unsafe behaviors.

## 6. Conclusions

This paper collected the investigation reports from hazardous chemical storage accidents that occurred in China during 2010–2019 in order to establish an improved HFACS model suitable for the analysis of hazardous chemical storage accidents. Through the improved HFACS model analysis, chi-square test and odds ratio analysis, we obtain the frequency of each accident cause and the causal relationships among the four levels of factors in the improved HFACS model. The conclusions are given as follows:

1. A modified HFACS model was established for human factors analysis of hazardous chemical storage accidents. Some items of the original HFACS model were not fully applicable to the analysis of hazardous chemical storage accidents. Therefore, according to the actual situation and characteristics of the collected hazardous chemical storage accidents, an HFACS model suitable for the analysis of hazardous chemical storage accidents was established.
2. The high-frequency human factors in hazardous chemical storage accidents were obtained. According to the results, the high-frequency accident causes under the HFACS framework were Resource Management (88.095%), Violations (85.714%), Inadequate Supervision (76.190%), Organizational Process (73.810%), Technological Environment (69.048%) and Personal Readiness (64.286%).
3. In total, 16 groups of significant causal relationships were determined among the four levels of factors in the improved HFACS model. The chi-square test and odds ratio analysis verified 16 groups of significant causal relationships among the four levels of factors, including Resource Management and Inadequate Supervision, Organizational Process and Planned Inappropriate Operations, Planned Inappropriate Operations and Technological Environment, Inadequate Supervision and Physical/Mental Limitations, Technological Environment and Skill-Based Errors, Personal Readiness and Violations, among others.

## Figures and Tables

**Table 1:** Review of the main relevant information of the human factors analysis and classification system (HFACS).

Author	Industry/Field of Application	Method	Principal Results	Reference
Dekker	-	-	The HFACS model is the most powerful tool for human factors analysis of various accidents	[7]
Shappell	Aviation	HFACS analysis method	The majority of accidents were caused by the air crew and environment	[8]
Daramola	Aviation	HFACS analysis method	Supervision violation to crew resource management to decision errors was considered the most likely path to accidents	[9]
Michal et al.	Aviation	HFACS and STAMP analysis method	Confirmed the feasibility of the STAMP-HFACS analysis method	[10]
Rashid et al.	Helicopter maintenance	HFACS-ME analysis method	Proposed the HFACS-ME model for helicopter maintenance accidents and studied the survival rate of helicopter maintenance accidents and the distribution of accident severity	[11]
Patterson and Shappell	Coal mine	HFACS-MI analysis method	Skill-based errors are the most common unsafe behavior, with no significant difference between different types of mines	[12]
Chauvin et al.	Maritime transportation	The improved HFACS analysis method	Most collision accidents were caused by decision errors	[13]
Baysari et al.	Railway transportation	HFACS and TRACEr methods	Suggested the effectiveness of the HFACS and TRACEr methods, but each tool seems to ignore certain important factors related to the occurrence of errors	[14]
Cohen et al.	Medical science	HFACS-Healthcare methods	HFACS and Healthcare can be used to identify system weaknesses during surgery	[15]
Hale et al.	Architecture	HFACS analysis method	Deficiencies in planning and risk assessment, hardware design, purchase and installation, and contracting strategies during building construction were found	[16]
Gong and Fan	Chemical industry	HFACS analysis method	Classified the human factors that led to the accident, confirming the usefulness and feasibility of the HFACS for accident analysis in the chemical industry	[17]
Zhou et al.	Chemical industry	HFACS analysis method	The interaction between different levels of human factors in the Ruihai company led to the accident, and the accident investigation report displayed limitations in the identification of human factors and guidance for similar accident prevention	[18]

**Table 2:** Calculation of the chi-square (*χ*^2^) value: 2 × 2 contingency table.

Low-Level Factors	High-Level Factors		Row Sum
	Exist	None	
Exist	*n*_11_(*f*_11_)	*n*_12_(*f*_12_)	*n_r_*_1_
None	*n*_21_*(f*_21_)	*n*_22_*(f*_22_)	*n_r_*_2_
Column Sum	*n_c_*_1_	*n_c_*_2_	*n*

**Table 3:** Details of 42 hazardous chemical storage accidents.

No.	Date of Accident	Enterprises	Type of Accident	Number of Deaths	Economic Damage (RMB)
1	7 January 2010	PETROCHINA Lanzhou Petrochemical Company	Explosion	6	9 million
2	29 June 2010	PETROCHINA Liaoyang Petrochemical Company	Explosion	3	1.5 million
3	24 October 2010	Dalian CNPC International Storage and Transportation Co., Ltd.	Fire disaster	-	-
4	18 January 2011	Inner Mongolia Wuhai Chemical Industry Co., Ltd.	Explosion	3	3 million
5	5 August 2011	Harbin Kaile Chemical Products Factory	Explosion	3	-
6	28 February 2012	Hebei KEEPER Chemical Industries Co., Ltd.	Explosion	25	44.59 million
7	1 March 2013	Jianping Hongshen Trading Co., Ltd.	Explosion	7	12.1 million
8	2 June 2013	PETROCHINA Dalian Petrochemical Company	Explosion	4	6.97 million
9	3 June 2013	Jilin Baoyuanfeng Poultry Co., Ltd.	Fire disaster	121	182 million
10	31 August 2013	Shanghai Wengpai Refrigeration Industry Co., Ltd.	Poisoning	15	25.1 million
11	14 September 2013	Fushun Shunte Chemical Co., Ltd.	Explosion	5	1.2 million
12	20 November 2013	Pucheng Xingzhen Xinglong Village Paper Tube Product Factory	Explosion	5	2 million
13	1 January 2014	Shandong Bin Yang gasification Co., Ltd.	Poisoning	4	5.36 million
14	21 March 2014	Inner Mongolia Baotou Steel Hefa Rare Erath Co., Ltd.	Explosion	1	-
15	6 April 2015	Tenglong Aromatics (Zhangzhou) Co., Ltd.	Explosion	-	94.57 million
16	16 July 2015	Shtar Science & Technology Group Petrochemical Co., Ltd.	Explosion	-	28.12 million
17	12 August 2015	Tianjin Port Ruihai International Logistics Co., Ltd.	Explosion	165	6.866 billion
18	28 November 2015	Handan Longgang Chemical Co., Ltd.	Poisoning	3	3.9 million
19	22 April 2016	Jiangsu Tak Bridge Company Limited Storage	Fire disaster	1	25.32 million
20	5 June 2016	Shandong Weifang Huahao Agrochemical Co., Ltd.	Poisoning	3	2.4 million
21	18 August 2016	Yangquan Coal Industry Group Taiyuan Chemical New Material Co., Ltd.	Explosion	-	1.75 million
22	8 September 2016	Shijiazhuang Jinzhou (illegal dye manufacturer)	Explosion	7	6.1 million
23	20 September 2016	Wanhua Chemical Group Co., Ltd.	Explosion	4	5.73 million
24	24 January 2017	Jiangxi Sanmei Chemical Co., Ltd.	Poisoning	2	7.4 million
25	27 February 2017	Jilin Songyuan Petrochemical Co., Ltd.	Explosion	3	5.9 million
26	2 April 2017	Anqing Wanhua Oil products Co., Ltd.	Explosion	5	7.866 million
27	13 May 2017	Hebei Lixing Special Rubber Co., Ltd.	Poisoning	2	3.2 million
28	5 June 2017	Linyi Jinyu Petrochemical Co., Ltd.	Explosion	10	44.68 million
29	28 September 2017	Guangdong Tenglong Chemical Technology Co., Ltd.	Fire disaster	-	247.6 thousand
30	19 December 2017	Shandong Rike Chemical Co., Ltd.	Fire disaster	7	14.79 million
31	10 February 2018	Jiujiang Zhongwei Technology Chemical Co., Ltd.	Explosion	2	1.7 million
32	1 March 2018	Tangshan Huayi IndustryHoldings Co., Ltd.	Fire disaster	4	5.37 million
33	27 March 2018	Zaozhuang Showers Industrial Co., Ltd.	Explosion	9	9 million
34	12 May 2018	Shanghai SECCO Petrochemical Co., Ltd.	Explosion	6	5.36 million
35	12 July 2018	Yibin Hengda Technology Co., Ltd.	Explosion	19	41.42 million
36	13 July 2018	Sichuan Jiangyou Changte No. 1 Factory Comprehensive Service Company	Explosion	1	1.4 million
37	12 November 2018	Jinan Huifeng Carbon Co., Ltd.	Explosion	6	11.45 million
38	28 November 2018	Hebei Shenghua Chemical Industry Co., Ltd.	Explosion	24	41.48 million
39	21 March 2019	Jiangsu Xiangshui Tianjiayi Chemical Co., Ltd.	Explosion	78	1986.35 million
40	15 April 2019	Qilu Tianhe Pharmaceutical Co., Ltd.	Poisoning	10	18.67 million
41	6 August 2019	Guangdong Guangkang Biochemical Technology Co., Ltd.	Fire disaster	-	9.5 million
42	16 September 2019	Guangzhou Human Engineering Materials Co., Ltd.	Explosion	2	2.26 million

Note: “-” indicates that no casualties or economic damage have been reported.

**Table 4:** Frequency and percentage of accident causes under the HFACS framework.

HFACS Framework Items		Frequency	Proportion (%)
Organizational Influences	Resource Management	37	88.095
	Organizational Climate	24	57.143
	Organizational Process	31	73.810
Unsafe Supervision	Inadequate Supervision	32	76.190
	Planned Inappropriate Operations	20	47.619
	Failure to Correct Problem	23	54.762
	Supervisory Violations	23	54.762
Preconditions for Unsafe Acts	Physical Environment	8	19.048
	Technological Environment	29	69.048
	Adverse Mental States	9	21.429
	Adverse Physiological States	1	2.381
	Physical/Mental Limitations	20	47.619
	Communication and Coordination	24	57.143
	Personal Readiness	27	64.286
Unsafe Acts	Skill-based Errors	20	47.619
	Decision Errors	22	52.381
	Perceptual Errors	3	7.143
	Violations	36	85.714

Note: Because there are multiple causes for the same accident, the sum of the percentages of accident causes under the HFACS framework is greater than 100%.

**Table 5:** Frequency statistics between Communication and Coordination and Decision Errors.

Decision Errors	Communication and Coordination		Row Sum
	Exist	None	
Exist	16	6	22
None	8	12	20
Column Sum	24	18	42

**Table 6:** *χ*^2^/*OR* value statistics between different levels of factors in HFACS (*p* < 0.05, *OR* > 1).

HFACS Level	*χ*^2^ Test		*OR*	95% Confidence Interval	
	*χ* ^2^	*p*		Upper Limit	Lower Limit
Causal Relationship Between Organizational Influence Level and Unsafe Supervision Level					
Resource Management × Inadequate Supervision	6.675	0.010	20.667	218.712	1.953
Organizational Climate × Inadequate Supervision	3.948	0.047	4.455	20.710	0.958
Organizational Climate × Supervisory Violations	9.259	0.002	7.800	31.151	1.953
Organizational Process × Planned Inappropriate Operations	5.177	0.023	6.231	33.771	1.150
Organizational Process × Supervisory Violations	4.546	0.033	4.848	22.107	1.063
Causal Relationship Between Unsafe Supervision Level and Preconditions for Unsafe Acts Level					
Inadequate Supervision × Physical/Mental Limitations	4.014	0.045	5.143	28.141	0.940
Inadequate Supervision × Communication and Coordination	7.394	0.007	8.800	49.162	1.575
Inadequate Supervision × Personal Readiness	11.212	0.001	14.286	83.171	2.454
Planned Inappropriate Operations × Technological Environment	4.546	0.033	4.722	20.887	1.068
Planned Inappropriate Operations × Communication and Coordination	4.972	0.026	4.333	16.248	1.156
Failure to Correct Problem × Technological Environment	4.375	0.036	4.275	17.420	1.049
Causal Relationship Between Preconditions for Unsafe Acts Level and Unsafe Acts Level					
Technological Environment × Skill-based Errors	7.843	0.005	9.000	48.437	1.672
Physical/Mental Limitations × Decision Errors	4.752	0.029	4.083	14.863	1.122
Communication and Coordination × Decision Errors	4.582	0.032	4.000	14.624	1.094
Personal Readiness × Skill-based Errors	4.107	0.043	4.000	15.868	1.008
Personal Readiness × Violations	4.706	0.030	13.000	125.520	1.346

Note: A × B indicates the causal relationship between cause A and result B.
